# The Cannabinoid Receptor Interacting Proteins 1 of zebrafish are not required for morphological development, viability or fertility

**DOI:** 10.1038/s41598-017-05017-5

**Published:** 2017-07-07

**Authors:** Laura Fin, Giorgia Bergamin, Roberto A. Steiner, Simon M. Hughes

**Affiliations:** Randall Division of Cell and Molecular Biophysics, New Hunt’s House, Guy’s Campus, King’s College London, London, SE1 1UL UK

## Abstract

The Cannabinoid Receptor Interacting Protein 1 (Cnrip1) was discovered as an interactor with the intracellular region of Cannabinoid Receptor 1 (CB1R, also known as Cnr1 or CB1). Functional assays in mouse show cannabinoid sensitivity changes and Cnrip1 has recently been suggested to control eye development in *Xenopus laevis*. Two Cnrip1 genes are described in zebrafish, *cnrip1a* and *cnrip1b*. *In situ* mRNA hybridisation revealed accumulation of mRNA encoding each gene primarily in brain and spinal cord, but also elsewhere. For example, *cnrip1b* is expressed in forming skeletal muscle. CRISPR/Cas9 genome editing generated predicted null mutations in *cnrip1a* and *cnrip1b*. Each mutation triggered nonsense-mediated decay of the respective mRNA transcript. No morphological or behavioural phenotype was observed in either mutant. Moreover, fish lacking both Cnrip1a and Cnrip1b both maternally and zygotically are viable and fertile and no phenotype has so far been detected despite strong evolutionary conservation over at least 400 Myr.

## Introduction

The discovery of (−)-*trans*-Δ^9^-tetrahydrocannabinol (THC) as the major psychoactive component of marijuana (*Cannabis sativa*)^1^ and subsequent cloning of the THC receptor^2^ helped to identify the endogenous cannabinoid system (eCS). The eCS is a signalling network responding to endogenous lipid transmitters (endocannabinoids) that bind to the G-protein coupled cannabinoid receptors CB1R and CB2R^3^. These receptors act via G_αi/o_ binding to their second and third intracellular loops and C-terminal 8^th^ helix to initiate signalling events typical of this class of transducing proteins, such as inhibition of adenylyl cyclase and activation of MAPK and FAK^4, 5^. CB1R has also been suggested to affect PI3K and PKB signalling and a variety of ion channels^3, 6^. Endocannabinoids are frequently produced post-synaptically in response to neurotransmitter signalling and act retrogradely on pre-synaptic terminals to attenuate pre-synaptic depolarisation and diminish further neurotransmitter release, forming a negative feedback loop^3, 7^. The eCS is involved in numerous pathological conditions, ranging from neurological disorders, such as Parkinson’s, Huntington’s and Alzheimer’s diseases^8^, to gastrointestinal, cardiovascular and reproductive disorders^9^ and is also potentially involved in relief of pain, chemotherapy-induced nausea and vomiting, and anorexia^10^. It thus has potential for therapeutic drug development^11^.

**Figure 1:**
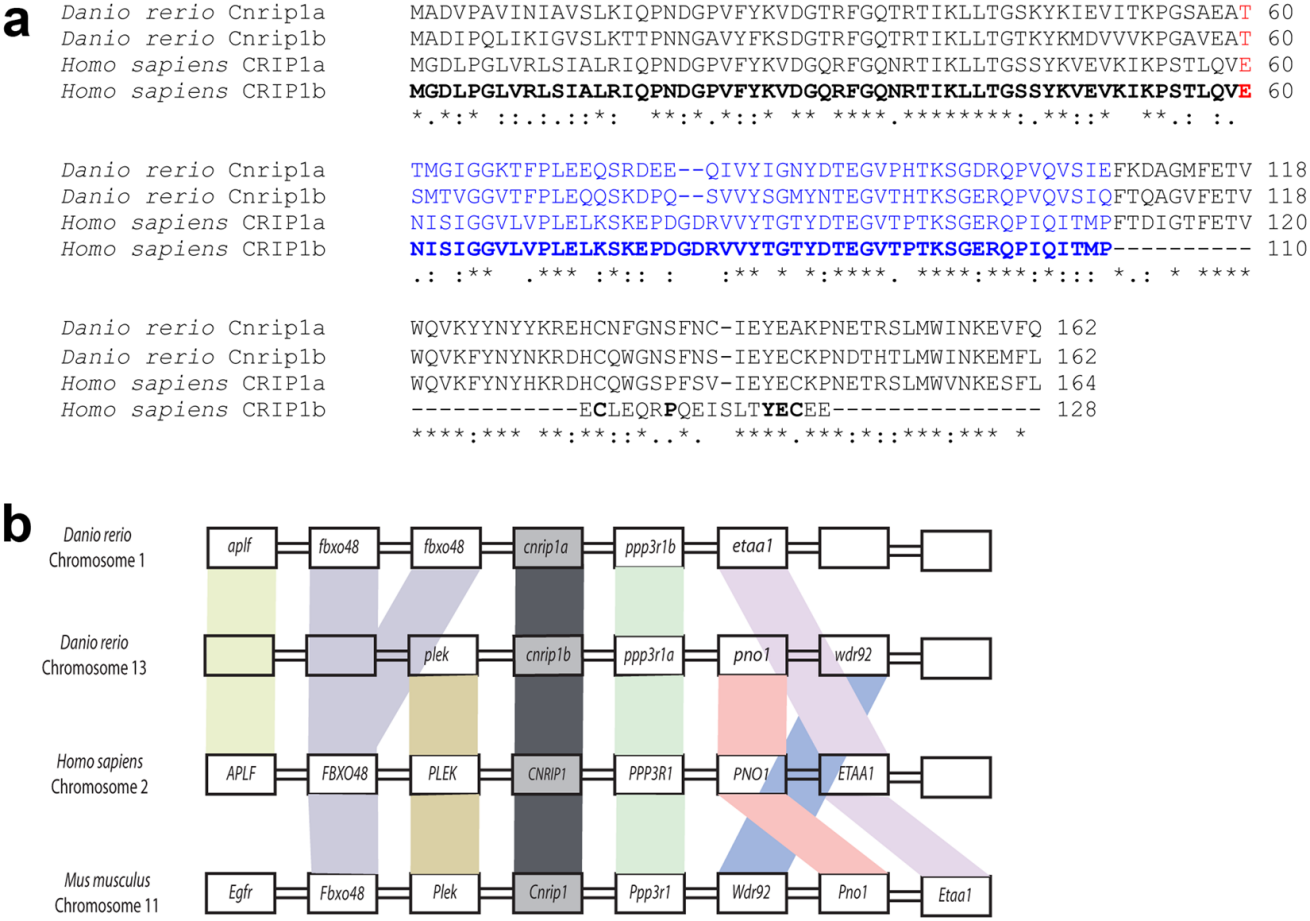
Cnrip1 genes in zebrafish. (**a**) Amino acid sequence alignment of Cnrip1a and Cnrip1b with the two splice variants of the single human *CNRIP1* gene, named CRIP1a and CRIP1b. Distinct exons are shown in alternating black and blue font (red indicates amino acids coded by two exons). Comparing both zebrafish genes and CRIP1a asterisk indicates identity and colon similarity between all three, full stop similarity between any two. Bold indicates identity between CRIP1a and CRIP1b. (**b**) Synteny of adjacent genes in vertebrate *CNRIP1* loci. Colours link homologous genes.

To understand the biochemical mechanism of endocannabinoid action and find possible drug targets, screens have been performed for proteins interacting with CB1R^12–14^. Two alternative splicing variant proteins, named CRIP1a and CRIP1b, deriving from the human *Cannabinoid Receptor Interacting Protein 1* gene (*CNRIP1*)^12^ have been reported to bind to the conserved C-terminal intracellular portion of CB1R, a region thought to be critical for receptor activation, sensitivity and recycling^15, 16^. Human CRIP1a and CRIP1b share identical N-termini, but diverge after residue 110 (Fig. 1a). The longer CRIP1a isoform (164 amino acids in human) is conserved across vertebrates and invertebrates, whereas CRIP1b (128 amino acids in human) is restricted to certain primates^17^. Functionally, CRIP1a, but not CRIP1b, has been reported to modulate CB1R-mediated tonic inhibition of voltage-gated Ca^2 + ^channels^12^ and recent evidence has indicated that CRIP1a can compete with β-arrestins for the C-terminal region of CB1R, suggesting a role of internalization of the receptor^18^.

Neuronal activity, endocannabinoid and D_2_ dopamine receptor (D_2_R) signalling can all regulate *CNRIP1* expression^19–21^. Over-expression of *CNRIP1* in rat dorsal striatum led to reduced ERK phosphorylation, decrease in transcript levels of certain opioid peptide precursors and the up-regulation of the opioid receptor DOR1 at both mRNA and protein level, mimicking effects observed when either CB1R or D_2_R were knocked-down^21^. Further over-expression studies have implicated CNRIP1 in CB1R signalling and turnover^18, 22–24^. Knockdown of CNRIP1 protein in a neuronal cell line can increase plasma membrane CB1R, alter G-protein subtype preference, augment basal phosphor-ERK level and enhance ERK phosphorylation in response to the CB1R agonist CP55940, but not to certain other CB1R agonists^25^. These data suggest the hypothesis that a role of *CNRIP1* is modulation of endocannabinoid signalling through CB1R. An important observation, however, is that whereas *CB1R* is primarily expressed in neurons of the central and peripheral nervous systems^26–30^, *CNRIP1* is expressed more broadly (http://www.ebi.ac.uk/gxa). Western blot analyses confirm that mouse CNRIP1 proteins are present in brain but also widely in heart, lung, kidney, intestine, testis, spleen, liver and muscle^12^. Association of *CNRIP1* promoter hypermethylation with colorectal and liver cancer and lymphoma has suggested the gene is a promising biomarker and may function in non-neural tissues^31–34^. Antisense morpholino knockdown of *Cnrip1* in Xenopus has been reported to cause brain and also eye defects early in development^35^, even though the eye lacks abundant Cnrip1 and CB1R in adult mouse^36^. Moreover, a *CNRIP1* gene is found in species that lack *CB1R*, such as the diploblast sea anemone *Nematostella vectensis* ^17^. Thus, CNRIP1 may have other functions independent of CB1R.

Here, we report the analysis of CNRIP1 expression and function in the zebrafish. Zebrafish have duplicate *cnrip1a* and *cnrip1b* genes expressed widely during early development. CRISPR/Cas9-mediated mutation of each gene leads to viable and fertile fish with no obvious phenotype. Double mutant animals are also viable and fertile, showing that CNRIP1 proteins are not essential for life.

## Results

### Zebrafish Cnrip1 genes

Two genes encoding Cnrip1 homologues were discovered in the zebrafish genome (Zv10). *Cnrip1a*, located on Chromosome 1, has three coding exons predicting a 162 amino acid protein with, respectively, 60% and 59% identity with the 164 amino acids of human CRIP1a and mouse CNRIP1. The region of greatest divergence was in the central region of the predicted proteins. The second gene, *cnrip1b*, is located on Chromosome 13, also has three exons and 162 amino acids with 69% identity with Cnrip1a and 59% identity to mammalian CNRIP1 (Fig. 1a). Identity between the two zebrafish paralogue cDNAs was 72% in the coding region, yielding 93% similarity at the amino acid level, with differences primarily in their N terminal 28 amino acids. Analysis in GeneTree (ensemble.org) shows that each gene clusters next to a cavefish orthologue as the sister groups to euteleost *cnrip1a* and *cnrip1b* clusters, consistent with the known teleost-specific genome duplication^37^. These groups cluster together and branch basally from the single amniote/amphibian *CNRIP1* gene cluster, following known evolutionary relationships.

Synteny analysis confirms the homology of both zebrafish genes with *CNRIP1* of human (Fig. 1b). Human and mouse genes have *PPP3R1*, *PNO1*, *WDR92*, *CD1* and *ETAA1* genes downstream and *PLEK*, *FBXO48* and *APLF* genes upstream. Zebrafish *cnrip1a* has *ppp3r1b* and *etaa1* downstream and two *fbxo48* homologues and *aplf* upstream. *Cnrip1b* has *ppp3r1a*, *pno1*, *wdr92* downstream and *plek* upstream. Thus, almost all flanking genes are conserved across ≥ 400 Myr of evolution, suggesting conserved regulation.

Although human *CNRIP1* RNA can be alternatively spliced, yielding proteins named CRIP1a and CRIP1b^12^ (Fig. 1a), no evidence of alternative splicing of either zebrafish gene was apparent in ENSEMBL. BLAST searches with the human alternate third exon of CRIP1b failed to identify sequences on zebrafish Chromosome 1 or 13. Indeed, neither zebrafish gene had significant homology with the alternate third exon of CRIP1b. This suggests that sub-functionalisation of an ancestral alternatively-spliced gene into two genes each of which encodes one splice variant does not explain the evolutionary retention of two *Cnrip1* genes in teleosts. *Cnrip1a* and *cnrip1b* have 24% and 28% identity to the last three exons of a *C. elegans* orthologue, *F29A7.4*, which has two additional 5′ exons encoding an N-terminal extension. Taken together, these data indicate strong conservation of structure, regulation and probably function of CNRIP1 across the vertebrates and possibly beyond.

### Expression of *cnrip1a* and *cnrip1b* transcripts

**Figure 2:**
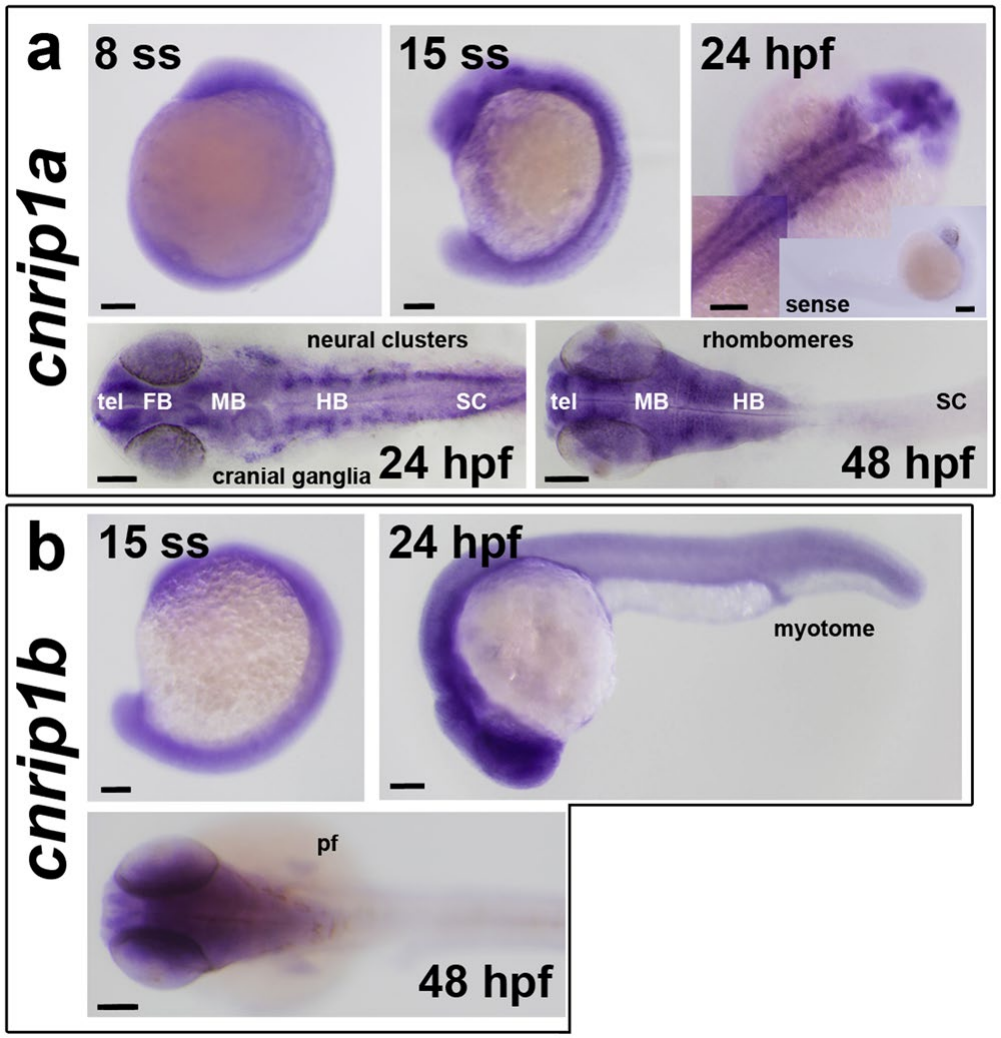
Accumulation of mRNAs from *cnrip1a* and *cnrip1b* genes. Whole mount *in situ* mRNA hybridisation of embryos at the indicated stages for antisense probes to *cnrip1a* (**a**) and *cnrip1b* (**b**). Lateral views (first two images in each panel) are anterior to top dorsal to left, except 24 hpf in which anterior is to left and dorsal to top. Dorsal views (remaining images) are anterior to left flatmounts (**a**) or wholemount (**b**), except panel a top right, which is a wholemount montage with anterior to top right. Sense control in inset in panel **a** top right is anterior to right dorsal to bottom. Quantitative evidence of reproducibility is given in Table S1. tel telencephalon, FB forebrain, MB midbrain, HB hindbrain, SC spinal cord, pf pectoral fin. Bars = 100 µm.

To analyse the expression pattern of *cnrip1a* and *cnrip1b* genes in zebrafish, embryos at several developmental stages were subjected to whole mount *in situ* hybridisation (Fig. 2; quantitative evidence of reproducibility for all experiments is presented in Table S1). *Cnrip1a* mRNA was detected throughout the embryo at around 10 hpf (Fig. 2a; Table S1). Hybridisation with a *cnrip1a* sense probe, in contrast, showed no significant staining. Consistent with a previous report^38^, at 24 and 48 hpf, *cnrip1a* was most highly expressed in the brain and spinal cord (Fig. 2a; Table S1). At 24 hpf, *cnrip1a* shows strongest expression in telencephalon, anterior and post-optic commissure, pineal gland, retinal ganglion cells, other head ganglia and neuron clusters in the hindbrain and in spinal cord (Fig. 2a; Table S1). At 48 hpf, the brain is widely stained. Olfactory receptors appear to be stained flanking the telencephalon. In hindbrain, *cnrip1a* mRNA is most abundant in radial glia on the sides of the rhombomeres. Signal in radial neuronal cells in the retina around the lens was suggestive of retinal ganglion cells. The spinal cord appeared less strongly stained at 48 hpf compared to 24 hpf (Fig. 2a; Table S1), but when 48 hpf fish tails were stained overnight separately from heads, a pattern similar to the one observed at 24 hpf appeared (data not shown). In general, *cnrip1a* mRNA was detected in many regions where post-mitotic neurons were abundant.

Differential gene expression could provide initial information on the possible additional functions of Cnrip1b in other body regions. *Cnrip1b* mRNA appears at around 10 hpf and, at this developmental stage, the pattern is similar to the one observed for *cnrip1a* mRNA (Fig. 2b; Table S1). At 24 and 48 hpf, the expression of *cnrip1b* diverges from that of *cnrip1a*. *Cnrip1b* is expressed in the brain, the eyes and the spinal cord, but the staining remains diffuse, whereas *cnrip1a* mRNA showed a clear enrichment in regions of post-mitotic neurons concentration (Fig. 2a; Table S1). In addition, unlike *cnrip1a*, *cnrip1b* mRNA was detected in developing somites and pectoral fins (Fig. 2b; Table S1). Thus, *cnrip1b* is expressed somewhat differently from *cnrip1a*, suggesting it could have a distinct function.

### Mutagenesis of Cnrip1 genes

**Figure 3:**
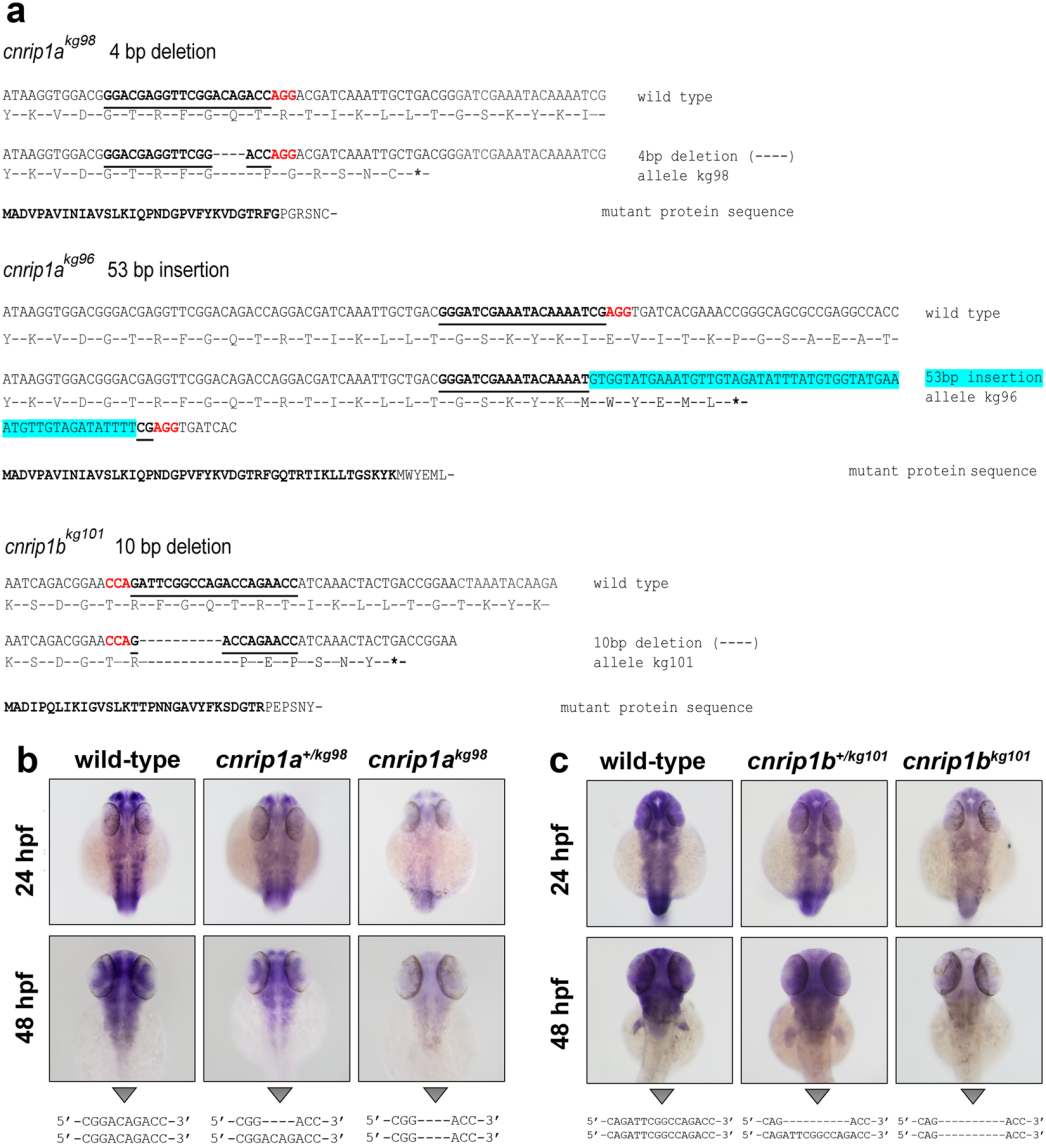
Mutagenesis of *cnrip1a* and *cnrip1b*. (**a**) Alignments of wild type with each of three mutant alleles showing the predicted expressed mutant polypeptide beneath. Bold underline indicates the gRNA target, red font the protospacer motif recognised by Cas9, hyphens deleted bases, blue highlight inserted bases and asterisks novel stop codons. In the mutant protein sequences, bold text indicates the residual wild type fragment and normal font the aberrant polypeptide tail. (**b**,**c**). To test for nonsense-mediated decay, about 50 siblings from in-crosses of *cnrip1a* ^*+/kg98*^ (**b**) and *cnrip1b* ^*+/kg101*^ (**c**) mutant carriers were subjected to *in situ* hybridisation for the cognate mRNA at the indicated stages, photographed, DNA extracted, PCR performed across the mutant locus and genotype confirmed by DNA sequencing as indicated beneath each panel. Quantitative evidence of reproducibility is given in Table S1.

Zebrafish mutants in *cnrip1a* and *cnrip1b* were generated using CRISPR/Cas9 genome editing (Fig. 3). Embryos from genotyped AB strain parents chosen to lack polymorphism in the target region were injected at 1-cell stage with gRNA and Cas9 RNA and these F0 fish outcrossed to similarly non-polymorphic wild-type AB to identify F1 fish heterozygous for mutations in each gene. Among 12 mutations obtained in one gRNA to *cnrip1a*, an allele *cnrip1a* ^*kg98*^ with a 4 bp deletion leading to a frameshift at amino acid 33 followed by in-frame stop codon after six further amino acids was selected (Fig. 3a). The novel stop codon in this allele is 20 amino acids ( = 60 bp) 5′ to the next in-frame methionine, making ribosomal re-initiation highly unlikely because the ribosome footprint on mRNA is only ~28 bp. A second allele, *cnrip1a* ^*kg96*^, was selected from three alleles produced with a distinct gRNA to *cnrip1a*; in this case a 53 bp insertion caused a frameshift at amino acid 47 followed by in-frame stop codon after six further amino acids (Fig. 3a). Beyond this stop codon at 7 and 19 bp downstream are two additional out of frame AUG codons which are themselves shortly followed by two additional stop codons, making in-frame re-initiation of the ribosome highly unlikely. For *cnrip1b*. among nine distinct mutations obtained with the single gRNA, a 10 bp deletion allele *cnrip1b* ^*kg101*^ was selected, which led to a frameshift at amino acid 31 followed by in-frame stop codon after six further amino acids (Fig. 3a). The nearest in-frame AUG codon is 40 bp downstream of the deletion, giving little possibility of ribosomal re-initiation.

For both genes, only mutations that inserted a stop codon as close as possible to the CRISPR target site were selected. In this way, only short truncated polypeptides are produced, which are typically unfolded and rapidly degraded. Moreover, as the mutant mRNAs lack translation beyond the first coding exon, they were degraded by the nonsense-mediated decay pathway^39^, as revealed by the greatly reduced *in situ* mRNA hybridisation signal observed in mutants (Fig. 3b,c; Table S1). Compared to wild type siblings, significantly less mRNA was also discerned in heterozygotes (Fig. 3b,c; Table S1).

Embryos homozygous mutant in each of the three alleles developed normally and no morphological abnormalities were detected (Fig. 3b,c; Table S1 and data not shown). In particular, eye morphology, pigmentation, lens and retina formation appeared normal (Fig. 3b,c; Table S1). From heterozygote in-crosses, an average of 20 embryos were genotyped at 48 hpf from three independent lays and χ^2^ tests showed no significant difference from expected Mendelian values (Table S1). Sibling larvae were again monitored at 5 dpf and weekly during growth in the nursery, but no consistent morphological or behavioural defects were observed in the predicted 25% of mutants (Table S1). Larvae were grown to adulthood and mutants (detected from finclip DNA) were observed at the expected 25% frequency and were indistinguishable from wild type and heterozygous siblings (Table S1). *Cnrip1a* ^*kg98*^ homozygous mutants survived as well as siblings until at least 19 months of age; *cnrip1b* ^*kg101*^ homozygous mutants survived until at least 16 months (Table S1). Both *cnrip1a* ^*kg98*^ and for *cnrip1b* ^*kg101*^ homozygous mutants were normally fertile and maternal/zygotic mutants (produced by breeding from a homozygous mutant mother) also showed no phenotype (Table S1), eliminating the possibility that Cnrip1 protein placed in the egg by a heterozygous mother supressed a more severe phenotype.

### Functional analysis of double mutants

**Figure 4:**
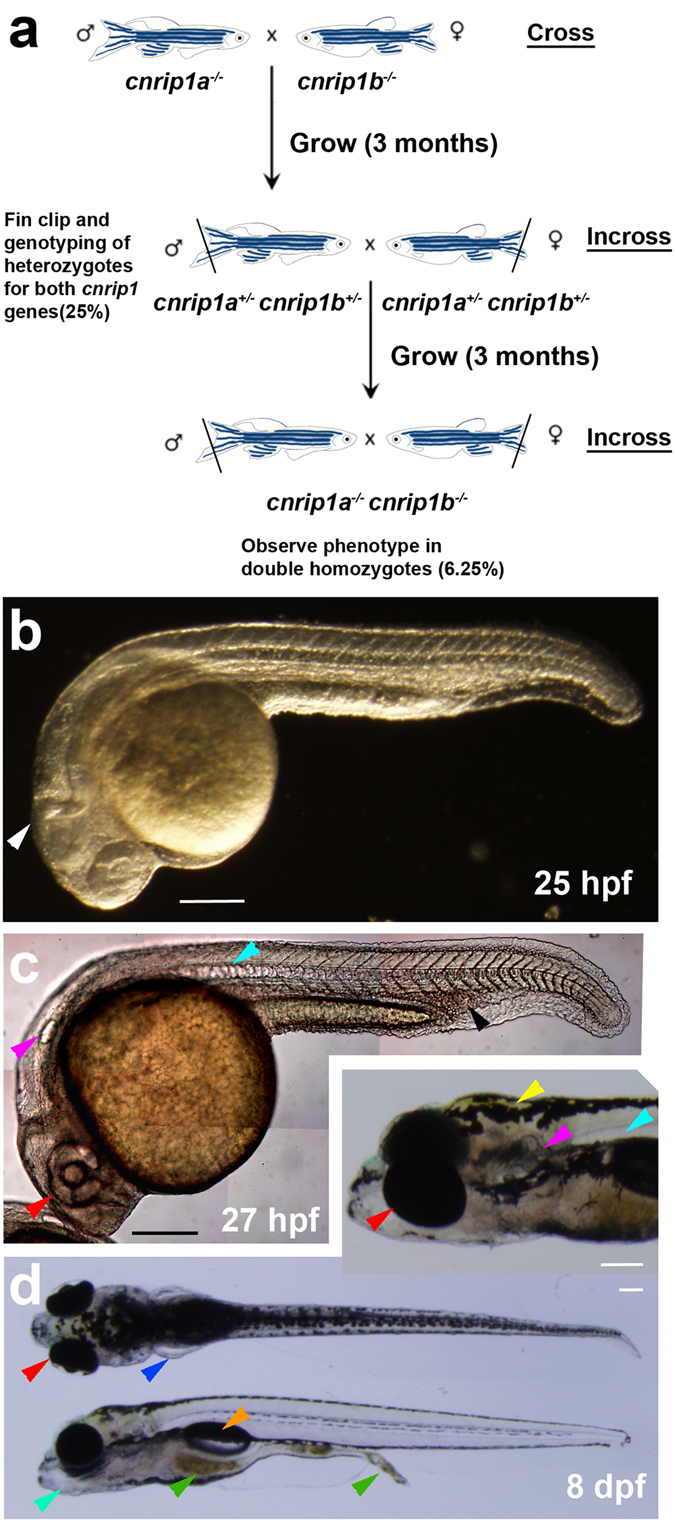
Maternal zygotic double *cnrip1a* ^*kg98*^ *;cnrip1b* ^*kg101*^ mutant fish develop normally. (**a**) Schematic of crosses to generate a *cnrip1* double mutant. (**b**) Darkfield image of 1 dpf double mutant indicating normal complex CNS folds (white arrowhead). (**c**) Differential interference contrast image of 1 dpf double mutant with arrowheads indicating normal eye (red), ear (purple), notochord (cyan) and haematopoetic tissue (black). (**d**) Dorsal, lateral and oblique (inset) views of 8 dpf larvae with arrowheads indicating pectoral fins (blue), jaw (turquoise), food traversing gut (green), swim bladder (orange) and xanthophores (yellow). Quantitative evidence of reproducibility is given in Table S1. All fish shown with anterior to left and dorsal up. Bars = 200 μm.

*Cnrip1a* ^*kg98*^ and *cnrip1b* ^*kg101*^ carriers were crossed to produce dual *cnrip1a* ^*kg98/+*^;*cnrip1b* ^*kg101/+*^ heterozygotes, which were in-crossed. Double mutants were observed at the expected frequency and again showed no morphological or behavioural defects (Fig. 4a; Table S1). Double mutants survived as well as their siblings until at least 15 months of age and, when in-crossed produced normal sized lays of healthy and viable larvae that grew to adulthood (Fig. 4b–d; Table S1). No defects in eye, brain, spinal cord, body shape and size, muscle, notochord, fins, pigmentation, swim bladder, pectoral fins, jaw, gut peristalsis, eating behaviour, eye movement, swimming, shoaling and mating behaviour or fertility were observed. Thus, insertion of early stop codons into both Cnrip1 genes of zebrafish yielded healthy and fertile adult fish.

## Discussion

The present work demonstrates that homozygous predicted loss of function mutations of all CNRIP1 genes in a vertebrate leads to viable and fertile organisms with no discernible characteristics different from wild type. *Cnrip1* expression is observed in the outer plexiform layer (OPL) of the mouse retina^36^ and morpholino loss of function studies have suggested that *Xenopus laevis cnrip1* regulates eye development^35^. However, we detected no eye defects in our maternal/zygotic *cnrip1a;cnrip1b* double mutants. Similarly, although Cnrip1 is expressed in mouse heart^12^, careful analysis of the developing heart revealed no defects and adult mutants did not show increased sudden cardiac death. Lastly, *CNRIP1* is highly expressed in the nervous system in species from mammals to fish, yet no morphological neural deficits or behavioural changes were observed in our mutants. These findings indicate that the function of the well-conserved CNRIP1 protein(s) confers a subtle increase in fitness that is not readily detected in the unchallenged aquarium environment.

There remains a formal possibility that our mutations, despite creating early stop codons in the first exon, are not null, either because the short truncated polypeptides can play some role or because of some kind of ribosomal re-initiation or stop codon read-through permits synthesis of a long protein. In the absence of functional antibodies recognizing the zebrafish proteins, we cannot be certain that no functional folded Cnrip1 protein is synthesised. However, we think this unlikely for several reasons. Firstly, nonsense mediated mRNA decay for each gene suggests that exon splice junction complexes are not removed downstream of the in-frame stop codons, indicating that ribosomes do not progress normally to the end of the three exon transcripts from each gene. Consistent with this view, there are no in-frame methionine codons nearby downstream of the novel stop codons that might encourage re-initiation by a stalled ribosome before it dissociates from the mRNA. Secondly, the reduced mRNA levels will mean that fewer ribosomal initiation events occur on each transcript and thus the absolute number of truncated polypeptides synthesised per unit time is likely lower than that of the full length proteins in wild types. Thirdly, the truncated polypeptides are under 40 amino acids in length (compared with 162 amino acids for each full length protein) and thus are unlikely to create a stable folded domain and will therefore be more rapidly degraded than the full length proteins. For these reasons, we suspect our mutants are functionally null.

Genomic analysis reveals that the genomes of zebrafish and most other fish contain duplicated copies of a clear homologue of the human *CNRIP1* gene. This strongly suggests that (a) *CNRIP1* is a conserved gene that has had an important function in vertebrates for at least 400 Myr, (b) that after the extra teleost genome duplication the duplicates developed essential functions sufficiently rapidly for both genes to be retained and well conserved. An invertebrate homologue of *CNRIP1*, *F29A7.4*, is present in *C. elegans*, has an ~80 amino acid N-terminal extension compared to vertebrates but is expressed in the ventral nerve cord and various neurons^40^. Akin to our findings in zebrafish, *F29A7.4* has not been observed to have a loss of function phenotype in two independent RNAi screens (http://www.wormbase.org/species/c_elegans/gene/WBGene00017914).

A consistent characteristic of *CNRIP1* family genes, also observed in zebrafish, is strong expression in neural tissue (recently reviewed^41^)﻿. Both *cnrip1a* and *cnrip1b* are expressed in the zebrafish nervous system, with *cnrip1a* mRNA sometimes concentrated in particular regions of neuronal terminal differentiation. Cells in specific regions of the brain and in placode-derived cranial ganglia express highly. This conserved location of expression is consistent with the conserved synteny of the chromosomal region around *CNRIP1* coding sequence across vertebrates. Expression in neurons is consistent with the suggested ability of CNRIP1 to bind to the intracellular region of CB1R, but does not exclude other functions in neurons. *Cnrip1b* is expressed more broadly than *cnrip1a*, appearing in muscle and fin tissue in the late embryo. As CB1R is expressed in many tissues, this finding could indicate functions beyond neuronal action of endocannabinoids. In future, it will be essential to assess the response of *cnrip1a* and *cnrip1b* mutant fish to gain and loss of function of endogenous and exogenous cannabinoids, as well as explore alternative functional avenues.

## Methods

### Zebrafish lines and maintenance

Genetically-altered *Danio rerio* on a primarily AB background were created and reared at King’s College London on a 14/10 hr light/dark cycle at 28.5 °C^42^. All experiments were performed in accordance with licences held under the UK Animals (Scientific Procedures) Act 1986 and later modifications and conforming to all relevant guidelines and regulations.

### *In situ* mRNA hybridisation

*In situ* mRNA hybridisation (ISH) was performed as described previously^43, 44^. IMAGE clones for both *cnrip1a* (clone ID: 7149263) and *cnrip1b* (clone ID: 8760081) genes (Thermo Scientific) were grown as in Table S2 and confirmed by sequencing. A PCR reaction was then performed to add T3 and T7 RNA polymerase sites to create antisense and sense probes. Generic pDNR-LIB primers were used for *cnrip1a*, while specific primers for *cnrip1b* were designed. Primers (Integrated DNA Technologies; Table S3) were used to amplify template DNA (Table S4), purified using the QIAquick PCR Purification Kit (QIAGEN) and digoxigenin-labelled RNA probes synthesised, treated with DNase and purified with the Illustra Microspin G-50 kit (GE Healthcare).

### CRISPR/Cas9 genome editing

Genome editing was adapted from ref. 45. CRISPR target sites in the first coding exon were selected with ZiFiT^46^ and potential off-targets minimized with the specific ZiFiT tool. Optimised flanking primers creating a ~120 bp PCR product for High Resolution Melt Analysis (HRM; 20 bp each, Tm 60 °C) and 200–400 bp product for DNA sequencing were selected for each gRNA with Primer 3 software^47^ and oligos bought from MWG Eurofins. CRISPR oligos were annealed, ligated into BsaI-digested pDR274 (Addgene), plasmid DNA purified, sequenced, digested with DraI and the 284 bp fragment gel-purified and used to synthesise gRNA with T7 RiboMAX large scale RNA production kit (Promega) which was phenol/ethanol purified, quantified by gel and Qubit (Invitrogen), aliquoted in 5 µl samples at 200 ng/µl and stored at −80 °C. NotI-HF-linearised pCS2-Cas9 was transcribed using mMessage mMachine SP6 kit (Ambion) and product purified as for gRNA.

HRMA-selected AB wild-type fish were DNA sequenced over the target loci to avoid polymorphisms, crossed and the resulting embryos injected with 1 nl containing 60 ng/µl gRNA, 200 ng/µl Cas9 mRNA and phenol red and GFP mRNA to select injected embryos. Twenty morphologically-normal 48 hpf larvae were analysed by HRMA to verify mutagenesis, their F0 siblings grown to adulthood and backcross F1 progeny analysed for transmission by HRMA. Mutant loci of F1s were sequenced to identify F0s transmitting mutations of interest, F1 siblings grown to adulthood and F1 heterozygotes identified by HRMA and sequencing of fin-clip DNA. Subsequent generations were bred by outcross to non-polymorphic wild-type AB. To generate double mutants, *cnrip1a* and *cnrip1b* F2 heterozygotes were crossed to obtain F3 progeny in which 25% of fish were dual heterozygotes (*cnrip1a* ^+/−^ *;cnrip1b* ^+/−^). These fish were in-crossed to generate a F4 progeny of which 6.25% were predicted to be homozygous for both genes (*cnrip1a* ^−/−^ *;cnrip1b* ^−/−^).

### High resolution melt analysis (HRMA)

Total genomic DNA was extracted from single embryos at 24 or 48 hpf or fin-clip tissue using 100 µl of PeqLab lysis solution (31-401-E) with 20 µg proteinase K incubated at 55 °C overnight and inactivated at 85 °C for 45 minutes. For the HRMA, 1 µl of each DNA lysate was mixed with 2× MeltDoctor HRM mix (Life Technologies), forward and reverse HRM primers (0.14 µM) to 32 µl and half loaded in duplicate wells on a MicroAmp Optical 384-well plate (Life Technologies), sealed with optical adhesive film, centrifuged at maximum speed for 30 seconds on a bench plate centrifuge to eliminate bubbles and HRMA performed on a ViiA7 real-time PCR system (Applied Biosystems).

### Screening for phenotype

Multiple lays of in-crosses between fish carrying identical or distinct alleles were screened for phenotypes. In each lay, about a hundred F2–F4 embryos at 4–10 hpf were monitored for alterations in gastrulation, epiboly and formation of the embryonic shield. From 10–36 hpf, defects in somites, myogenesis, brain, spinal cord, notochord, eye and heart were screened. Subsequently, alteration in brain, spinal cord, eye, heart, cardiovascular system and fin morphology, pigmentation hatching, touch response and swimming patterns were analysed. At 96 hpf, larvae were screened again for touch response and motility, defects in pigmentation, pectoral fins, jaw, branchial arches, blood, ear, liver and gut. At 5 dpf, larvae were transferred to the nursery, where they were monitored on a weekly basis for later developmental defects or behaviour abnormalities.

### Statistics

χ^2^ tests were performed in Excel taking p < 0.05 as significant.

## Electronic supplementary material

Supplementary Information
