# Supplementary material for: The Cannabinoid Receptor Interacting Proteins 1 of zebrafish are not required for morphological development, viability or fertility
Source: Sci Rep. 2017 Jul 7;7:4858. doi: 10.1038/s41598-017-05017-5 (PMC5501828; doi:10.1038/s41598-017-05017-5)
Supplement: Supplementary file 1 — Supplementary Information [file 41598_2017_5017_MOESM1_ESM.pdf]

SUPPLEMENTARY DATA for

The Cannabinoid Receptor Interacting Proteins 1 of zebrafish are not required for morphological development, viability or fertility.

Laura Fin, Giorgia Bergamin, Roberto A. Steiner and Simon M. Hughes*

Randall Division of Cell and Molecular Biophysics, New Hunt's House, Guy's Campus, King's College London, London SE1 1UL UK.

Figure S1

Position of sequencing and HRM primers and upstream gRNA on *cnrip1a*:

```
1      TATGCTCTGGATTACGGTATGGGTGTGATAATGTGTGATGATGACGTCACGGCATCTCTCAAACAGCTTCCTGGGAGCAT
1      ATACGAGACCTAATGCCATACCCACACTATTACACACTACTACTGCAGTGCCGTAGAGAGTTTGTCGAAGGACCCTCGTA

                                                                               M  A  D  V
81     CGGTCCTGCATCCGTCGAGTGACACCGGGAGCCCTGGAAACACACCGACCTCCGTTATCCAGCAGTCATGGCTGACGTTC
81     GCCAGGACGTAGGCAGCTCACTGTGGCCCTCGGGACCTTTGTGTGGCTGGAGGCAATAGGTCGTCAGTACCGACTGCAAG

       P  A  V  I  N  I  A  V  S  L  K  I  Q  P  N  D  G  P  V  F  Y  K  V  D  G  T  R
161    CCGCGGTAATAAACATCGCCGTTTCGTTGAAAATCCAGCCCAATGACGGACCCGTGTTTTATAAGGTGGACGGGACGAGG
161    GGCGCCATTATTTGTAGCGGCAAAGCAACTTTTAGGTCGGGTTACTGCCTGGGCACAAAATATTCCACCTGCCCTGCTCC    2

       F  G  Q  T  R  T  I  K  L  L  T  G  S  K  Y  K  I  E  V  I  T  K  P  G  S  A  E
241    TTCGGACAGACCAGGACGATCAAATTGCTGACGGGATCGAAATACAAAATCGAGGTGATCACGAAACCGGGCAGCGCCGA
241    AAGCCTGTCTGGTCCTGCTAGTTTAACGACTGCCCTAGCTTTATGTTTTAGCTCCACTAGTGCTTTGGCCCGTCGCGGCT    3

        A
321    GGCCACGTAAGTCCGTCTGATATTGGATTTCTTTTACAATGTAAATAATATGTTTGTCATTAGAAACAAGGTCGAAAGGT
321    CCGGTGCATTCAGGCAGACTATAACCTAAAGAAAATGTTACATTTATTATACAAACAGTAATCTTTGTTCCAGCTTTCCA

401    GCACCCTGCCTGATAAAATATATTACATAGATACAGTATATACACGTAAGAAATATGTACA   461
401    CGTGGGACGGACTATTTTATATAATGTATCTATGTCATATATGTGCATTCTTTATACATGT   461
```

Position of sequencing and HRM primers and downstream gRNA on *cnrip1a* map:

```
1    TATGCTCTGGATTACGGTATGGGTGTGATAATGTGTGATGATGACGTCACGGCATCTCTCAAACAGCTTCCTGGGAGCAT
1    ATACGAGACCTAATGCCATACCCACACTATTACACACTACTACTGCAGTGCCGTAGAGAGTTTGTCGAAGGACCCTCGTA

                                                                                 M  A  D  V
81   CGGTCCTGCATCCGTCGAGTGACACCGGGAGCCCTGGAAACACACCGACCTCCGTTATCCAGCAGTCATGGCTGACGTTC
81   GCCAGGACGTAGGCAGCTCACTGTGGCCCTCGGGACCTTTGTGTGGCTGGAGGCAATAGGTCGTCAGTACCGACTGCAAG

      P  A  V  I  N  I  A  V  S  L  K  I  Q  P  N  D  G  P  V  F  Y  K  V  D  G  T  R
161  CCGCGGTAATAAACATCGCCGTTTCGTTGAAAATCCAGCCCAATGACGGACCCGTGTTTTATAAGGTGGACGGGACGAGG
161  GGCGCCATTATTTGTAGCGGCAAAGCAACTTTTAGGTCGGGTTACTGCCTGGGCACAAAATATTCCACCTGCCCTGCTCC

      F  G  Q  T  R  T  I  K  L  L  T  G  S  K  Y  K  I  E  V  I  T  K  P  G  S  A  E
241  TTCGGACAGACCAGGACGATCAAATTGCTGACGGGATCGAAATACAAAATCGAGGTGATCACGAAACCGGGCAGCGCCGA
241  AAGCCTGTCTGGTCCTGCTAGTTTAACGACTGCCCTAGCTTTATGTTTTAGCTCCACTAGTGCTTTGGCCCGTCGCGGCT

      A
321  GGCCACGTAAGTCCGTCTGATATTGGATTTCTTTTACAATGTAAATAATATGTTTGTCATTAGAAACAAGGTCGAAAGGT
321  CCGGTGCATTCAGGCAGACTATAACCTAAAGAAAATGTTACATTTATTATACAAACAGTAATCTTTGTTCCAGCTTTCCA

401   GCACCCTGCCTGATAAAATATATTACATAGATACAGTATATACACGTAAGAAATATGTACA   461
401   CGTGGGACGGACTATTTTATATAATGTATCTATGTCATATATGTGCATTCTTTATACATGT   461
```

Position of sequencing and HRM primers and gRNA on *cnrip1b* map:

```
1    GGGATTATTACTCGGGAACAGGAACCGCACACATGACTTTAGACCTGCTTTCTGCGATGCCGTTTTTTCACGGTTTTCCA
1    CCCTAATAATGAGCCCTTGTCCTTGGCGTGTGTACTGAAATCTGGACGAAAGACGCTACGGCAAAAAAGTGCCAAAAGGT

                                                                                     M
81  CCGAATCGCGCTTTGCTTCAGCCGGGCAGATCTTGAGCTGATTCTCCACACCTGACTGCCTTCATCTTCTTCTTCATCAT
81  GGCTTAGCGCGAAACGAAGTCGGCCCGTCTAGAACTCGACTAAGAGGTGTGGACTGACGGAAGTAGAAGAAGAAGTAGTA

       A  D  I  P  Q  L  I  K  I  G  V  S  L  K  T  T  P  N  N  G  A  V  Y  F  K  S
161  GGCTGACATCCCGCAGCTCATTAAAATCGGCGTTTCGCTGAAAACCACACCGAATAACGGAGCCGTTTACTTCAAATCAG
161  CCGACTGTAGGGCGTCGAGTAATTTTAGCCGCAAAGCGACTTTTGGTGTGGCTTATTGCCTCGGCAAATGAAGTTTAGTC

      D  G  T  R  F  G  Q  T  R  T  I  K  L
241  ACGGAACCAGATTCGGCCAGACCAGAACCATCAAACTACTGACCGGAACTAAATACAAGATGGATGTGGTGGTCAAACCG
241  TGCCTTGGTCTAAGCCGGTCTGGTCTTGGTAGTTTGATGACTGGCCTTGATTTATGTTCTACCTACACCACCAGTTTGGC

321  GGAGCAGTGGAGGCGACGTGAGTGAATTAATGATCATTTGTTTATGTGTAAGTGAAATGATAGACTATCTTTGATTATAT  400
321  CCTCGTCACCTCCGCTGCACTCACTTAATTACTAGTAAACAAATACACATTCACTTTACTATCTGATAGAAACTAATATA  400
```

Fig. S1. Position of primers and gRNAs on *cnrip1a* and *cnripb*. Position of CRISPR target sites on the exon 1 and intron 1 of *cnrip1a* and *cnrip1b*, sequences and features of sequencing and HRM primers for each chosen CRISPR target site and their positions on the respective gene sequences. Accession numbers: *cnrip1a* (GenBank AN: NM_001003607.2; Uniprot AN: Q6DBX3), *cnrip1b* (GenBank AN: XM_679802.4; Uniprot AN: E9QJH9). For each gene the amino acid sequence in bold corresponds to the translated region of the first exon of the gene. gRNAs are highlighted and primers underlined and colour coded in pairs.

Table S1. Quantitative evidence for reproducibility of results

Figure Number and panel or Experiment	Wild-type	Heterozygote sibling	Mutant sibling
2a 8ss	10 + 15	n/a	n/a
2a 15ss	10 + 12	n/a	n/a
2a 24hpf wholemount<br>flatmount	30 + 20 + 20<br>5 + 6	n/a<br>n/a	n/a<br>n/a
2a 48hpf	30 + 15 + 15	n/a	n/a
2b 15ss	10 + 10	n/a	n/a
2a 24hpf	30 + 25 + 14	n/a	n/a
2a 48hpf	20 + 10 + 15	n/a	n/a
3b 24hpf	8/42 (19%)	24/42 (57%)	10/42 (24%)
3b 48hpf	14/53 (26%)	27/53 (51%)	12/53 (23%) χ^2 = 0.54
3c 24hpf	10/36 (28%)	19/36 (53%)	7/36 (19%)
3c 48hpf	11/40 (27.5%)	21/40 (52.5%)	8/40 (20%) χ^2 = 0.56
cnrip1a$^{kg98/+}$ incross survival (63 + 140 + 28)/ (67 + 157 + 30) (91%)	(3 + 3 + 6)/ (15 + 20 + 20) (22%)	(9 + 11+ 10)/ (15 + 20 + 20) (55%)	(4 + 5 + 3)/ (15 + 20 + 20) (22%) χ^2 = 0.78
cnrip1a$^{kg96/+}$ incross survival (54 + 107 + 80)/ (57 + 124+ 89) (89%)	(5 + 8 + 4)/ (20 + 25 + 15) (28%)	(11 + 12 + 8)/ (20 + 25 + 15) (52%)	(4 + 5 + 3)/ (20 + 25 + 15) (20%) χ^2 = 0.64
cnrip1b$^{kg101/+}$ incross survival (105 + 52 + 86)/ (109 + 65+ 92) (91%)	(7 + 5 + 4)/ (25 + 15 + 20) (27%)	(13 + 7 + 12)/ (25 + 15 + 20) (53%)	(5 + 3 + 4)/ (25 + 15 + 20) (20%) χ^2 = 0.67
cnrip1a$^{kg98/+}$ incross morphogenesis	0/50 (Mutants found at 6 months at 11/31 (χ^2 = 0.17))		
cnrip1a$^{kg96/+}$ incross morphogenesis	0/50 (Not assessed at adults)		
cnrip1b$^{kg101/+}$ incross morphogenesis	0/50 (Mutants found at 4 months at 1/13 (χ^2 = 0.15))		
cnrip1a$^{kg98/kg98}$ MZ incross	n/a	n/a	50/50 normal to 5 dpf
cnrip1b$^{kg101/kg101}$ MZ incross	n/a	n/a	50/50 normal to 5 dpf
cnrip1a$^{kg98/+}$;*cnrip1b*$^{kg101/+}$ incross survival at 11 months: (all ~100 appear wild-type)	cnrip1a+/+;cnrip1b+/+, +/+;+/-, +/+;-/-, +/-;+/+, +/-;+/-, +/-;-/-, -/-;+/+, -/-;+/-, -/-;-/-<br>Genotyped 33: 0, 3, 2, 5, 9, 5, 1, 6 , 2<br>Expected: 2.06 4.12 2.06 4.12 8.25 4.12 2.06 4.12 2.06<br>χ^2 = 0.84 (Both male and female double mutants still breeding at 18 months)		
4b morphology	n/a	n/a	10/10
4c morphology	n/a	n/a	10/10
4b,c behaviour	n/a	n/a	67/67
4d morphology	n/a	n/a	10/10 + 2/2
4d behaviour	n/a	n/a	45/45
4d survival to 5 dpf	n/a	n/a	67/67 + 45/45
4d survival to 3 months	n/a	n/a	40/54 (75%)

Double numbers separated by '+' indicate replicate experiments on separate lays.
2a 8ss - number of embryos with not-spatially-restricted expression.
2a 15ss - number of embryos with not-spatially-restricted expression and concentration of signal in hindbrain spots and ventral regions.
2a 24 hpf- number of embryos with elevated expression in bilateral neural clusters in telencephalon, midbrain, hindbrain and spinal cord and lateral expression in cranial ganglia.
2a 48 hpf - number of embryos with most intense signal in brain, weak expression in retina and little expression in pectoral fin bud, trunk and tail.
2b 15ss - number of embryos with not-spatially-restricted expression.

2b 24 hpf- number of embryos with widespread expression and more intense signal in head and eyes.
2b 48 hpf - number of embryos with predominant cranial expression and weak pectoral fin bud expression.
3b,c - numbers of genotyped embryos with, respectively, strong (wild-type), medium (het) and weak (mutant) expression/total embryos analysed (%). X^2 test results are given for each mutant in rightmost column showing Mendelian frequencies.
Single mutant incross survival: Values in left column represent numbers of embryos surviving at 48 hpf out of total in each lay. Values in columns 2-4 represent numbers of embryos of each genotype out of total genotyped at 48 hpf in three separate lays. X^2 test result is given in rightmost column showing Mendelian frequencies. MZ = maternal zygotic mutants.
Single mutant incross morphogenesis: Values represent the number of affected embryos observed/ total screened daily until 5 dpf and weekly thereafter for consistent defects affecting brain, eye, heart or general body size and morphology and behaviour. The entire lay was not retrospectively genotyped, but mutants were found at expected Mendelian frequencies in genotyped individuals, as described in parantheses.
4b morphology - fraction of 25 hpf embryos observed under dark field that showed normal mid-hindbrain border fold, somite morphology, overall body shape, heart beat, eye size and shape.
4c morphology - fraction of 27 hpf embryos with normal eye size, retinal folding, lens size and position, ear size and morphology, somite number, chevron shape, size, morphology and apposition, notochord refractivity, size, regularity and vacuolation, red blood cell formation in ventral tail.
4b,c behaviour - fraction of 25-27 hpf embryos with spontaneous movements in chorion and response by movement to dish tap and/or poke of chorion with forceps, normal heartbeat and circulation through tail.
4d survival – fraction exiting chorion by 4 dpf
4d morphology - fraction of 8 dfp embryos with normal eye size, pigmentation, lens position and size, fin length and shape, ear morphology, size and presence of otic vesicles, melanophore and xanthophore frequency, distribution and shape, notochord structure, swim bladder inflation and jaw shape.
4d behaviour - fraction of 8 dpf embryos with normal swimming (spontaneous and response to tank tap), eye movements (spontaneous and response to shadow), fin burst movements (spontaneous), gut food content and peristalsis, air gulping to inflate swim bladder.

Table S2. IMAGE clones encoding *cnrip1a* and *cnrip1b*.

**Feature**	**IMAGE ID: 7149263**	**IMAGE ID: 8760081**
**cDNA coding for**	Cnrip1a	Cnrip1b
**Insert size**	850 bp	1015 bp
**Region covered**	50% 5'UTR. Coding exons 1-3. 30% 3'UTR	60% coding region of Exon 1. Coding exons 2+3. 350 bp 3'UTR.
**BLAT result**	3 regions of Chr 1 >99%	3 regions of Chr 13 >99%
**Restriction sites**	SfiIA and SfiIB	NotI and EcoRV

Table S3. **Primer pairs for RNA polymerase site insertion (ISH probes generation).**

Primer pairs	Gene
Fwd:<br>**TAATACGACTCACTATAGGGAGA**TGGATAACCGTATTACCGCC<br>Rev:<br>CGCGCA**ATTAACCCTCACTAAAG**CACTAGTCATACCAGGATC	*cnrip1a*
Fwd:<br>**TAATACGACTCACTATAGGGAGA**ACTCAGGCGGGTGTTTTC<br>Rev:<br>GGATCC**ATTAACCCTCACTAAAGGGA**ACTATAATAATTGATGTGCCGTGTC	*cnrip1b*

T7 (Fwd sense, negative control) and T3 (Rev antisense) consensus sequences are shown in bold font.

Table S4. **Reaction mixture composition and amplification cycle parameters for RNA polymerase site insertion.**

Reagent	Quantity	Reaction cycle
DNA template	50 ng	95°C for 4 minutes<br>95°C for 30 seconds ⎫<br>63°C for 30 seconds ⎬ 35 cycles<br>72°C for 2 minutes ⎭<br>72°C for 7 minutes
10X Reaction Buffer	2.5 µl	
dNTPs mix	0.4 µl (0.4 mM)	
Forward primer	1.25 µl (0.5 µM)	
Reverse primer	1.25 µl (0.5 µM)	
Vent DNA polymerase	0.5 µl (1 U)	
MilliQ water	to 25 µl	

Annealing temperature for primers (63°C) was calculated using the NEB Tm calculator (http://tmcalculator.neb.com).
